# Mining the Wheat Grain Proteome

**DOI:** 10.3390/ijms23020713

**Published:** 2022-01-10

**Authors:** Delphine Vincent, AnhDuyen Bui, Doris Ram, Vilnis Ezernieks, Frank Bedon, Joe Panozzo, Pankaj Maharjan, Simone Rochfort, Hans Daetwyler, Matthew Hayden

**Affiliations:** 1 Agriculture Victoria, AgriBio, Centre for AgriBioscience, 5 Ring Road, Bundoora, VIC 3083, Australia; AnhDuyen.Bui@agriculture.vic.gov.au (A.B.); Doris.Ram@agriculture.vic.gov.au (D.R.); Vilnis.Ezernieks@agriculture.vic.gov.au (V.E.); Simone.Rochfort@agriculture.vic.gov.au (S.R.); Hans.Daetwyler@agriculture.vic.gov.au (H.D.); matthew.hayden@agriculture.vic.gov.au (M.H.); 2 Department of Animal, Plant and Soil Sciences, School of Life Sciences, La Trobe University, Bundoora, VIC 3083, Australia; f.bedon@latrobe.edu.au; 3 Agriculture Research Victoria, 110 Natimuk Road, Horsham, VIC 3400, Australia; Joe.Panozzo@agriculture.vic.gov.au (J.P.); pankaj.maharjan@agriculture.vic.gov.au (P.M.); 4 Centre for Agricultural Innovation, University of Melbourne, Parkville, VIC 3010, Australia; 5 School of Applied Systems Biology, La Trobe University, Bundoora, VIC 3083, Australia

**Keywords:** *Triticum aestivum*, shotgun proteomics, LC–MS/MS, protease, normalisation, data mining

## Abstract

Bread wheat is the most widely cultivated crop worldwide, used in the production of food products and a feed source for animals. Selection tools that can be applied early in the breeding cycle are needed to accelerate genetic gain for increased wheat production while maintaining or improving grain quality if demand from human population growth is to be fulfilled. Proteomics screening assays of wheat flour can assist breeders to select the best performing breeding lines and discard the worst lines. In this study, we optimised a robust LC–MS shotgun quantitative proteomics method to screen thousands of wheat genotypes. Using 6 cultivars and 4 replicates, we tested 3 resuspension ratios (50, 25, and 17 µL/mg), 2 extraction buffers (with urea or guanidine-hydrochloride), 3 sets of proteases (chymotrypsin, Glu-C, and trypsin/Lys-C), and multiple LC settings. Protein identifications by LC–MS/MS were used to select the best parameters. A total 8738 wheat proteins were identified. The best method was validated on an independent set of 96 cultivars and peptides quantities were normalised using sample weights, an internal standard, and quality controls. Data mining tools found particularly useful to explore the flour proteome are presented (UniProt Retrieve/ID mapping tool, KEGG, AgriGO, REVIGO, and Pathway Tools).

## 1. Introduction

Contributing about 20% of the total calories consumed by humans, wheat (*Triticum aestivum* L.) is the most cultivated crop worldwide. Wheat offers not only a wide adaptability and high yield potentials, but also contains gluten proteins whose viscoelastic properties allow dough to be turned into bread and other food products such as pasta and noodles [1]. Sustaining wheat production and quality with reduced agrochemical inputs and developing new varieties with enhanced quality for specific end-uses are the main objectives addressed by breeding programs [1].

There is an ongoing requirement for wheat research and breeding to accelerate genetic gain to increase wheat yield while maintaining or improving grain quality traits if the demands of human population growth are to be met [2]. Efficient breeding and germplasm section strategies must be underpinned by functional annotations of the whole genome. Owing to the large size of wheat polyploid genome, containing more than 85% of repetitive DNA, sequencing efforts have lagged behind other major cereals. The whole genome sequence was finally completed in 2018 following international collaborative efforts spanning over a decade [2,3]. The annotation of gene models paved the way for ‘omics’ studies, which can accelerate breeding with rapid and robust screening assays applied to large-scale experiments using high-throughput technologies. Proteomics screening assays of wheat flour can assist breeders in selecting the best performing genotypes and filtering out the worst performing ones.

Advances in wheat genomics, transcriptomics, and metabolomics have been highlighted [4]. Wheat proteomics publications have steadily accumulated since the new millennium (PubMed timeline with the keywords ’wheat proteome*’) and peaked the year the wheat genome sequence was published. Some advances on the topic have been reviewed [5,6]. Combining several omics technologies can provide a more complete molecular view of biological systems. Using transcriptomics and iTRAQ-labelling proteomics to probe the early stages of wheat grain development, Yang and colleagues quantified and identified 85,000 genes and 7500 proteins [7]. Zhang and colleagues used deep proteome and metabolome analyses to shed light on the wheat grain filling process, where they dissected grains into seed coat, embryo, endosperm, and cavity fluid during major developmental stages to reveal the dynamic accumulation patterns of molecules over space and time [8]. 

Two-dimensional polyacrylamide gel electrophoresis (2-D PAGE) or two-dimensional electrophoresis (2-DE) [9] has traditionally been used as a standard procedure for proteomics research. Several groups have resorted to 2-DE to explore wheat grain proteomes [10,11,12,13,14,15,16]. Gel-based technologies are labour intensive and time consuming and therefore not suitable for large numbers of samples. Mass spectrometry (MS) is the most popular tool to identify, characterise, and quantify proteins and their proteoforms since it offers high throughput and can be applied to large sample numbers [17]. This highly resolving technology separates ionised molecules based on their mass to charge ratio (*m*/*z*). Most mass spectrometers used for proteomics are quadrupole time-of-flight, ion trap, and orbitrap systems and are compatible with a high-performance liquid chromatography (HPLC) system placed online at the front-end. Most popular LC systems in proteomics apply reverse phase (RP) conditions which separate molecules based on their hydrophobicity and retention time (RT). RPLC–MS not only helps separate compounds bearing identical *m*/*z* that cannot be differentiated by MS alone, but also allows for the detection of low-abundance molecules by separating them from higher abundance ones [8]. Shotgun bottom-up or peptide-centric proteomics is the most common gel-free approach in which whole protein samples are digested into peptides prior to LC–MS/MS analysis. Some applications of these techniques to probe wheat grain development and filling [7,8] compare old and modern germplasm [18], or detect allergens [12]. 

LC–MS workflows are automated, fast, flexible, adaptable, and durable, hence lend themselves perfectly to high-throughput proteomics. In an ambitious project aiming at screening flour proteomes from thousands of wheat lines, our first step was to develop a robust analytical method. To this end, we optimised various steps in the process, including the amount of flour, type of extraction buffer and protease, as well as main LC parameters. We performed LC–MS/MS experiments to identify wheat proteins in order to retain methods yielding the greatest number of protein identities. The best method was validated using 96 wheat cultivars. We mined the thousands of identified proteins using online tools such as KEGG, Gene Ontology, and Pathway Tools.

## 2. Results and Discussion

**Figure 1:**
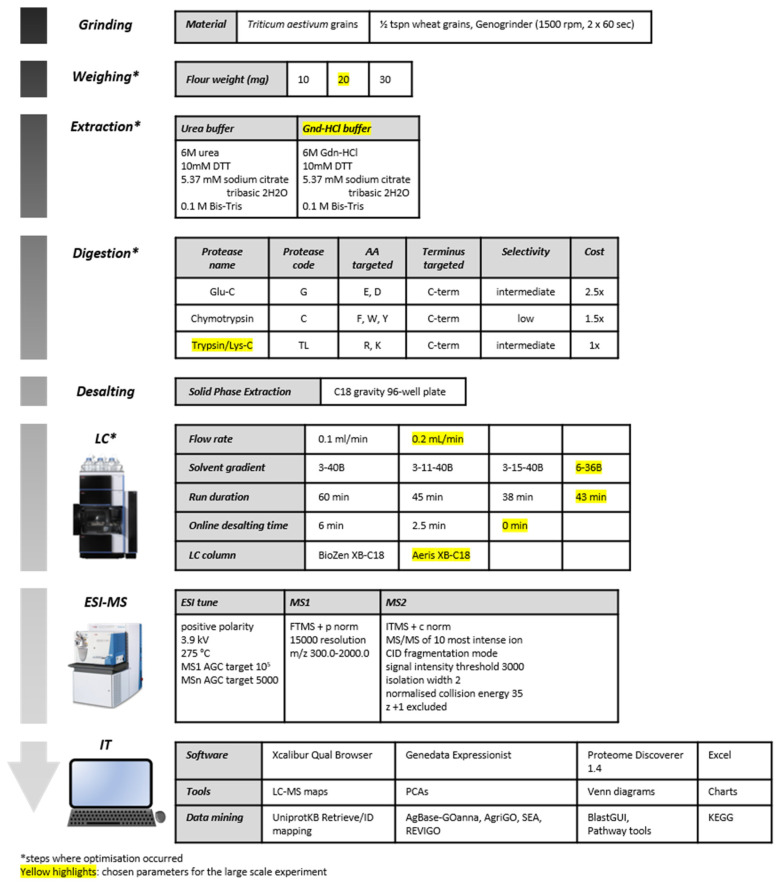
Experimental design. The asterisk denotes where technical optimisation occurred, and the yellow highlights indicate which parameters were selected for the large-scale experiment.

We developed a label-free, gel-free quantitation method to analyse the proteome of wheat grain using LC–MS and LC–MS/MS. This high throughput method is suitable for processing thousands of samples and does not compromise LC–MS peak resolution. The experimental design used to develop this optimised method is shown in Figure 1. The wheat cultivars used are listed in Supplementary Table S1. 

The following sections present and discuss each optimised steps of the workflow, the method validation, and protein identification results.

### 2.1. Testing Flour Weights

Sample pulverisation was one of the major bottlenecks of the sample preparation workflow. We used a Geno/Grinder which accommodated 36 grinding jars simultaneously and allowed us to grind 100 samples in about one hour. Using several Geno/Grinders would increase the throughput. Following pulverisation of the wheat grains into fine flour, 3 weights (10, 20, and 30 mg) were tested for extraction of the proteins with 0.5 mL guanidine hydrochloride (Gnd-HCl) buffer, using 3 cultivars and 4 technical replicates. This corresponded to volume:powder ratios of 50, 25, and 17 µL/mg. Another bottleneck was achieving complete resuspension of the powder in a 0.5 mL volume of buffer. We have explored different options including extensive vortexing and incubation times using a sonicator bath that can accommodate more than one tube at a time. However, we failed to achieve total and consistent solubilisation of the flour, as could be seen from dry powder at the bottom of the tube and powder clumps in the buffer (data not shown). In our hands, only a sonicator probe could ensure fast and efficient homogenisation of the flour into a water-based buffer. This probe-sonication step was critical for reproducible flour resuspension and only added 30 s to the workflow. We have opted for a single probe which was affordable, but the reader is welcome to investigate multielement probes to increase the throughput.

**Figure 2:**
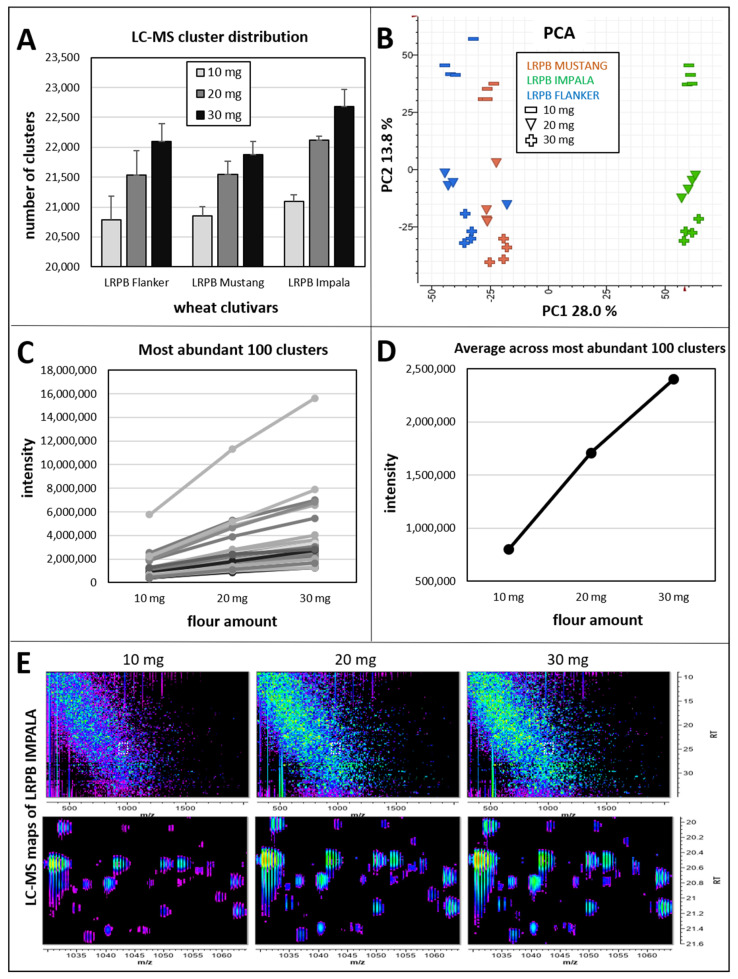
Testing flour weights. Three wheat cultivars (LRPB Mustang, LRPB Impala, and LRPB Flanker) were weighed in four replicates, extracted using 0.5 mL Gnd-HCl buffer, and 10 µL extract aliquots were digested using trypsin/Lys-C protease mixture. LC–MS data were acquired using LC method 5. (**A**) Histogram of the number of LC–MS clusters averaged per cultivar and flour amount; (**B**) PC1 vs. PC2 plot based on LC–MS quantitative data; (**C**) line chart of the 100 most abundant LC–MS clusters averaged across cultivars; (**D**) line chart of the averages of the 100 most abundant LC–MS clusters; (**E**) LC–MS maps of LRPB Impala cultivar for each of the flour amount tested with zoomed-in sections at 20–21.6 min and 1030–1070 *m*/*z*.

The number of LC–MS peaks observed ranged from 20,254 to 22,963, with an average of 21,622 (SD = 645). LC–MS isotopic peaks from the same peptide were grouped into a cluster by Genedata Refiner, which was used for quantitation purpose. The number of clusters was comparable between wheat cultivars and increased with the amount of flour used but tapered off when 30 mg (17 µL/mg) was used, indicating incomplete resuspension of the flour (Figure 2A). 

Principal component analysis (PCA) showed separation of the wheat cultivars along PC1 (28%) and gradual discrimination of the flour weights along PC2 (14%) (Figure 2B). Whilst PC2 clearly arranged flour amounts by increasing values (10, 20, 30 mg from top to bottom), the smallest amount (10 mg) was well separated from the heavier ones (20 and 30 mg) along this axis. High reproducibility was achieved as evidenced by all four replicates clustering together, except for a one outlier for a 20 mg replicate of LRPB Flanker. 

A line chart of the 100 most abundant LC–MS clusters averaged across varieties demonstrated increasing intensity as the flour amount increased (Figure 2C), albeit with the intensity of some clusters, notably the most abundant one (cluster_08661), dipping when 30 mg were extracted. This was even more evident when the 100 most prominent clusters were averaged per weight (Figure 2D), which suggested 17 µL/mg exceeded the limit of solubility for wheat flour. Indeed, 30 mg were difficult to resuspend and produced viscous extracts.

Complete LC–MS maps and zoomed-in sections showed that the number of LC–MS clusters and their intensity grew as more flour was solubilised (Figure 2E).

Based on these results, we chose an amount of 20 mg (25 µL/mg) over 10 mg as it yielded more data. Moreover, it was also quicker to weigh 20 mg with 1% accuracy than 10 mg and therefore would better suit large-scale experiments that require high throughput. We confirmed the reproducibility of this weight using six wheat cultivars, each with four replicates (Supplementary Figure S1). Weighing was another major bottleneck in our sample preparation workflow. An option to increase the throughput would be to invest in an automatic weighing platform such as the Flex Swile (Chemspeed Technologies, Füllinsdorf, Switzerland).

When optimising the resuspension of a pulverised sample in a buffer, one can either vary the amount of powder weighed or vary the volume of solution used. We chose the former and kept the buffer volume constant. What matters ultimately is using the best ratio powder:solution to ensure complete solubilisation of the sample and guarantee reproducible protein extraction across samples. If the aim of a study is to maximise the number of proteins without trying to compare their quantities, then increasing the amount of flour would augment the number of proteins recovered and identified, albeit at the cost of reproducibility and thus preventing any quantitative analysis. Our study aimed at creating a quantitative method dictated by high reproducibility via consistent protein extraction across samples. In our hands, the best compromise was with 20 mg of flour in 0.5 mL buffer.

The powder:volume ratio that produced the best results during our tests was 25 µL/mg, same ratio used by [19,20]. Usage of more concentrated ratios are often reported in the literature: 4 µL/mg (50 mg: 200 µL) [13]; 5 µL/mg (100 mg:0.5 mL) [21]; 6.7 µL/mg (30 mg:200 µL) [8]; 10 µL/mg [10,11,15,18,22,23,24]; 16 µL/mg (50 mg:800 µL or 100 mg:1.6 mL) [14,25]; and 20 µL/mg (0.5 g:10 mL) [26], although less frequently, more diluted ratios are mentioned: 33 µL/mg (30 mg:1 mL) [27]; 40 µL/mg; (100 mg:4 mL) [28]; 50 µL/mg (300 mg:15 mL) [29]; and 100 µL/mg (10 mg:1 mL) [30]. Our optimal ratio fitted within the 4 to 100 µL/mg range reported in published literature.

### 2.2. Testing Extraction Buffers

Two extraction buffers differing only in the chaotrope reagent they contained (urea or Gnd-HCl) were tested on three cultivars and four replicates. LC–MS/MS data were acquired for 60 min.

**Figure 3:**
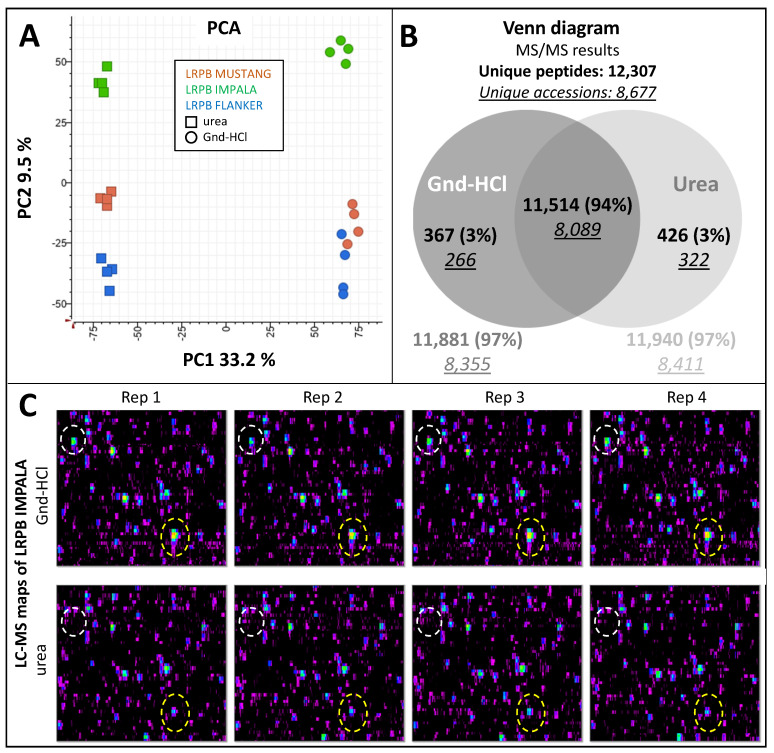
Testing extraction buffers. Twenty milligrams (±0.2 mg) from three wheat cultivars (LRPB Mustang, LRPB Impala, LRPB Flanker) was weighed in four replicates, extracted using 0.5 mL Gnd-HCl or urea buffer. Protein extracts were assayed to obtain protein concentrations and 100 µg proteins were digested using trypsin/Lys-C protease mixture. LC–MS/MS data were acquired using LC method 1. (**A**) PC1 vs. PC2 plot based on LC–MS quantitative data; (**B**) Venn diagram of the identified unique peptides and accessions for each extraction buffer; (**C**) zoomed-in section of LC–MS maps at 28–36 min and 950–990 *m*/*z* of LRPB Impala cultivar for both extraction buffers tested across four technical replicates; cluster qualitative and quantitative differences are highlighted in ovals.

PCA analysis of the LC–MS clusters clearly discriminated the samples according to the buffer used along PC1 (33%) and by cultivar along PC2 (9%). The technical replicates grouped together confirming good reproducibility (Figure 3A). 

A total of 12,307 peptides were identified in the samples, which matched 8677 unique *T. aestivum* protein accessions. A Venn diagram indicated both buffers gave comparable identification rates, with the Gnd-HCl and urea samples yielding 11,881 (97%) and 11,940 (97%) identified peptides, respectively (Figure 3B), which corresponded to 8355 and 8411 accessions. Most peptides were observed across both buffers (11,514; 94%), with only 3% uniquely recovered by each extraction solution. MS/MS identification results are listed in Supplementary Table S2.

The LC–MS zoomed-in maps showed both extraction methods provided excellent reproducibility and produced very similar patterns (Figure 3C) with a few quantitative differences (exemplified by ovals in Figure 3C). Based on these observations, both buffers could be used for shotgun proteomics. We selected Gnd-HCl as it is cheaper, which is an important factor for large-scale experiments.

A number of extraction methods are reported in published literature. Bose and colleagues compared a urea-based buffer and a Tris-HCl buffer to recover proteins from wheat flour followed by trypsin digestion and LC–MS/MS analysis. Both buffers yielded comparable identification rates, with a slight advantage to urea (8846 peptides; 1483 accessions) relative to Tris-HCl (8632 peptides; 1405 accessions); 79% of the accessions were shared across both buffers [23]. Other reports have employed the sequential extraction method devised by Osborne in 1924 [31] to recovery successive protein fractions using first an aqueous buffer to extract albumins, second a salt solution to retrieve globulins, third an ethanol/water mixture to solubilise gliadins, and finally a propanol solution to extract glutenins [8,10,12,15,19,20,21,22,24,26,27,29,30,32]. Another multistep procedure involved an initial phenol/ammonium acetate phase partition followed by solvent precipitation and Tris-HCl buffer resuspension [7,8,18,33]. A complex protocol employed an initial solubilisation using potassium chloride followed by methanol/ammonium acetate precipitation and final resuspension in a urea buffer [13]. A variation of this was to solubilise wheat flour in a sodium dodecylsulfate buffer followed by precipitation using cold acetone and urea resuspension [25]. A shorter method skipped the initial solubilisation to directly precipitate flour protein in cold acetone with or without trichloroacetic acid and resuspend it in urea [11,14]. A sucrose fractionation to isolate cell walls followed by solubilisation of the proteins in an acetate buffer with CaCl_2_ or LiCl was employed to analyse the proteome of endosperm and outer layers of developing seeds [34,35]. A particularly complex method applying Tris/glycerol precipitation followed by urea resuspension of the pellet then reprecipitation in cold acetone and final redissolution in urea was performed to recover proteins from various wheat organs including maturing grains [36]. 

Whilst very effective for in-depth proteomics analyses of wheat grain subproteomes such as gluten proteins, cell wall proteins or endosperm/embryo proteins over developmental periods, elaborate extraction protocols cannot be used in large-scale studies as the multiple steps involved would be too time consuming and labour intensive. Therefore, we developed a fast single-step protein extraction for our screening assay. Urea has often been used in gel-based proteomics, notably for 2-DE experiments as it is compatible with the isoelectric focusing separation stage. Many of the works cited above have employed 2-DE to analyse wheat flour proteins, hence the ample use of urea. To our knowledge, Gnd-HCl has not been employed to extract proteins from wheat grains. We have found it very efficient at recovering and denaturing proteins for shotgun proteomics on various species, plants and animals alike [37,38,39,40,41,42,43], and have proven its superiority over urea in *Cannabis sativa* buds [42].

### 2.3. Testing Proteases

Three digestions of wheat proteins were tested using orthogonal proteases: Glu-C which cleaves the negatively charged amino acid (AA) residues E and D [44], chymotrypsin which targets the hydrophobic AAs Y, F, and W, and a mixture of trypsin-Lys-C which cleaves the positively charged AA residues R and K. The particulars of these proteases, including their specificity, their complementarity, and how together they improve plant protein coverage have been thoroughly demonstrated and discussed in Vincent et al. 2019 and 2020 [40,43]. The proteases were tested on three cultivars with four replicates. LC–MS/MS data were acquired for 60 min using LC method 1.

**Figure 4:**
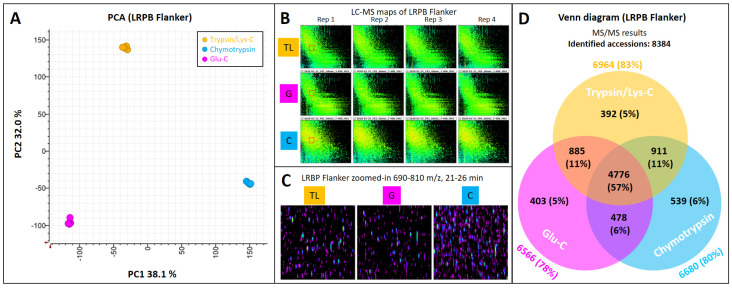
Testing proteases. Twenty milligrams (±0.2 mg) from three wheat cultivars (LRPB Mustang, LRPB Impala, LRPB Flanker) was weighed in four replicates, extracted using 0.5 mL Gnd-HCl buffer. Protein extracts were assayed to obtain protein concentrations and 100 µg proteins were digested using chymotrypsin, Glu-C, or trypsin/Lys-C proteases. LC–MS/MS data were acquired using LC method 1. (**A**) PC1 vs. PC2 plot based on LC–MS quantitative data; (**B**) LC–MS maps of LRPB Flanker cultivar for each of the proteases tested across four technical replicates, boxed sections are zoomed-in in panel C; (**C**) zoomed-in section of LC–MS maps at 21–26 min and 690–810 *m*/*z* to highlight cluster qualitative and quantitative differences; (**D**) Venn diagram of the identified accessions for each protease. TL, trypsin/Lys-C; G, Glu-C; C, chymotrypsin.

PCA analysis confirmed the orthogonality of the three protease treatments, evidenced by the triangular pattern observed in plot of the first two principal components (Figure 4A). PC1 (38%) separated chymotrypsin from Glu-C and trypsin/Lys-C, while PC2 (32%) isolated trypsin/Lys-C from the other two proteases.

LC–MS maps showed trypsin/Lys-C and Glu-C produced more similar pattern than chymotrypsin (Figure 4B), and zoomed-in sections revealed more LC–MS clusters when chymotrypsin was used (Figure 4C).

A total of 8384 accessions were identified in this dataset. A Venn diagram exemplified the overlap of identities across the three sets of proteases (Figure 4D). Trypsin/Lys-C identified the largest number of protein accessions (6964; 83%) and produced 392 (5%) trypsin/Lys-C-specific accessions. Chymotrypsin identified 6680 (80%) accessions and generated 539 (6%) chymotrypsin-specific accessions. Glu-C generated the smallest number of identified accessions (6566; 78%) and Glu-C-specific proteins (403; 5%). The overlap across all three proteases was 57% (4776 protein accessions). Chymotrypsin and trypsin/Lys-C shared 911 (11%) identities, Glu-C and trypsin/Lys-C shared 885 (11%) accessions and chymotrypsin and Glu-C shared 478 (6%) identifications.

Our results demonstrated that targeting distinct AA residues via orthogonal proteases increased proteome coverage. However, this was only feasible for a small-scale experiment as resorting to multiple proteases did incur significant costs. As trypsin/Lys-C was the cheaper protease (Figure 1), we chose it for our future large-scale analyses. Despite the commercialisation of numerous enzymes, serine protease trypsin remains the gold standard in proteomics and as such is the most commonly used enzyme. Trypsin‘s leading position can be attributed to its commercialisation at affordable cost, high efficiency, cleavage-site specificity, reliability and production of peptides amenable to MS. The lower cleavage efficiency of trypsin towards K than R residues can be mitigated by combining Lys-C, which specifically cleaves at the carboxyl terminus of K residues and operates under the same conditions as trypsin. We took advantage of this in our study. 

Trypsin has had wide usage in wheat grain proteomics. Many scientists have employed a gel-based strategy [10,11,12,13,14,15,16] or a gel-free approach [7,8,12,18] combined with the digestion of proteins from excised 2-D spots or whole extracts using trypsin for peptide sequencing purposes. Using 2-DE to study early wheat growth events, Wong and colleagues identified 26 unique accessions, indicating that mobilisation of the starch reserves during germination and seedling development was underpinned by increased protease activity and protein reduction by thioredoxin [13]. Also using 2-DE to observe the early processes of wheat grain formation, Nadaud and colleagues identified 249 unique accessions, including proteins involved in primary metabolisms, proteins associated with starch granules, and heat shock proteins (HSPs) [11]. Yang and colleagues found that high temperature and water stress applied during early seed formation induced changes in 65 protein accessions involved in primary metabolism and storage and stress response; HSPs and 14-3-3 proteins were only affected by high temperatures [15]. With 33 identities from 2-D gel spots, Garcia-Molina and colleagues showed that low gliadin transgenic wheat lines compensated by accumulating high molecular weight (HMW) glutenins [10]. Xue and colleagues studied the effect of applying split nitrogen fertilisation at critical wheat growth stages using 2-DE and reported the differential expression of 19 storage protein accessions [14]. Using a label-free nLC–MS/MS workflow on flour samples from old and modern wheat landraces, Di Francesco and colleagues showed that 59% of the 671 accessions identified were shared across all genotypes [18]. Yang and colleagues employed an iTRAQ-labelling nLC–MS/MS strategy to identify 3600 proteins accumulating during early grain development, including 306 development stage-specific proteins [33]. They revisited this topic recently and the 7500 identified accessions belonged to carbohydrate metabolism, amino acid metabolism, lipid metabolism, and cofactor, as well as vitamin metabolism [7]. As can be seen in the aforementioned works, gel-free approaches yielded far more identities than gel-based experiments, except for the study by Zhang and colleagues who adopted an SDS-PAGE shotgun strategy to analyse in depth the proteomes of seed coat, embryo, endosperm, and cavity fluid during the grain filling process [8]. Of the 15,484 accessions identified, many were involved in starch synthesis such as sucrose synthases, starch phosphorylase, granule-bound and soluble starch synthase, pyruvate phosphate dikinase, and 14-3-3 proteins, together with sugar precursors. 

The prolamin storage proteins of wheat seeds contain little lysine and arginine content, and therefore are not particularly amenable to tryptic digestion. By cleaving Y, F, W, and to a lesser extent L, chymotrypsin was identified as a suitable alternative protease to trypsin [19]. Chymotrypsin is less specific than trypsin as it targets multiple AAs. This serine protease cleaves hydrophobic residues such as Y, F, and W and under certain conditions L and M. Consequently, chymotrypsin generates peptides which cover a proteome space orthogonal to that of trypsin [45]. Adopting a gel-free workflow, Fiedler and colleagues digested gluten proteins independently with trypsin and chymotrypsin to discover novel peptide biomarkers that can be used for gluten detection in commercial gluten-free flour [30]. They reported that more peptides were generated when chymotrypsin was used than with trypsin. Following 2-DE separation, Dupont and colleagues employed three proteases: trypsin, thermolysin, and chymotrypsin, to sequence 157 wheat flour proteins, including glutenins, gliadins, farinins, purinins, triticins, globulins, and alpha-amylase inhibitors [25,46]. Thermolysin is a metalloproteinase that cleaves the N-terminus of hydrophobic residues L, F, V, I, A, and M under high temperatures [47]. Thermolysin is completely orthogonal to trypsin but not to chymotrypsin, since they both target F, L, and M. Following a 2-DE separation, 49 protein spots were excised and digested using trypsin, chymotrypsin, or thermolysin and revealed that nongluten fractions discriminated cultivars better than gluten fractions [16]. The aspartic protease pepsin, which exhibits a broad cleavage specificity but preferentially targets Y, F, and W residues (similar to chymotrypsin), was used by Prandi and colleagues to determine gluten peptide biomarkers [28].

Here, we chose to test three complementary protease sets: trypsin, chymotrypsin, and Glu-C. As far as we know, this is the first time Glu-C has been used on wheat grains. 

### 2.4. Testing LC Separation

Several LC parameters were tested, including total duration, solvent gradient, online desalting time, flow rate, and separation columns. To this end, six LC methods were devised (described in Section 3.2.3). 

**Figure 5:**
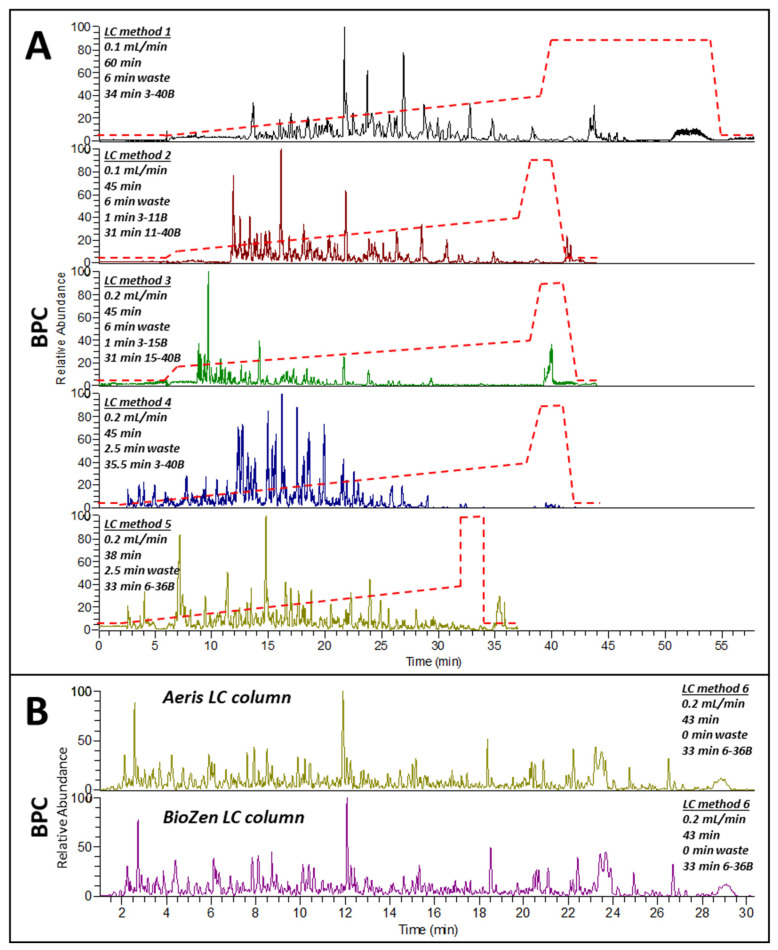
Testing LC separation. Twenty milligrams (±0.2 mg) from LRPB Flanker) was weighed, extracted using 0.5 mL Gnd-HCl buffer, and digested using trypsin/Lys-C. LC methods are described in the Materials and Methods. (**A**) BPCs obtained to test LC durations, solvent gradients, initial online desalting durations, and flow rates; red dotted lines depict the solvent gradient; (**B**) BPCs using the LC method 6 to compare BioZen and Aeris XB-C18 LC columns.

LC method 1 was 60 min long including an initial 6 min online desalting and a 3–40% solvent gradient for 34 min followed by a 10 min washing step at high solvent concentration; the flow rate was 0.1 mL/min. Peptides eluted from 13 min onward and displayed high peak resolution (Figure 5A, top panel). Whilst producing well-resolved base peak chromatograms (BPCs), this method was deemed too long for high sample throughput.

To shorten the separation time, the total duration of method 2 was dropped to 45 min by reducing the washing step to 2 min; the flow rate and desalting time were unchanged. To speed peptide elution, an initial steep increase from 3–11% solvent was introduced, followed by an 11–40% solvent gradient for 31 min. This accelerated peptide elution by 1.5 min (11.5 min onward) and peak resolution was negatively impacted (Figure 5A second panel).

To further accelerate peptide elution, a slight variation of LC method 2 was introduced by doubling the flow rate (0.2 mL/min) and applying a steeper 3–15% solvent initial gradient (LC method 3). This resulted in peptide elution 8.5 min earlier, but peak resolution was even more negatively affected (Figure 5A third panel).

To mitigate this, LC method 4 maintained a 0.2 mL/min flow rate but reduced the online desalting time to 2.5 min and applied a 3–40% solvent for 35.5 min. This resulted in an early peptide elution (2.7 min) and restored the high peak resolution (Figure 5A fourth panel). Yet not many peptides eluted past 29 min.

To remedy this, LC method 5 maintained a 0.2 mL/min flow rate and a 2.5 online desalting time but applied a 6–36% solvent gradient for 33 min with a total LC run duration of 38 min. This produced an early peptide elution (2.5 min), followed by sustained peptide elution until the end of the run, without compromising peak resolution (Figure 5A fifth panel).

While LC method 5 was optimal, it did not include a washing step long enough to ensure proper LC column regeneration. Furthermore, the initial online desalting step was unnecessary as the solid phase extraction (SPE)-cleaned digests were already desalted. We attempted the workflow schematized in Figure 1 without the SPE step to save both time and money, but the LC column became clogged after about 50 samples, despite the online desalting stage (data not shown). Hence, LC method 6 retained all the parameters of LC method 5 but eliminated the online desalting step and extended the washing step to 5 min for total run duration of 43 min. 

Using LC method 6, we compared two LC columns designed by Phenomenex for peptides separation: Aeris and bioZen. Both produced very similar chromatograms (Figure 5B). We selected bioZen for future use due to its lower cost.

Comparing our study to other published shotgun gel-free experiments, we found that, while RP-HPLC with a C18 separation column and mobile phases similar our ours were commonly used to separate peptides, column oven temperatures, flow rates, mobile phases, solvent gradients, and LC run durations varied. Oven temperatures ranged from room temperature [7,12,22], slightly warm (35 °C, [28,30]), to warm (50 °C, [18]). Our higher oven temperature of 60 °C helped accelerate peptide elution, which will be advantageous in high-throughput workflows. Reported flow rates depended on whether the HPLC system used accommodated nano-to-micro (nLC) or normal (UPLC) flow. Rates varied from 300 nL/min [18], 500 nL/min [30], 60 µL/min [22], 0.2 mL/min [7,28], to 0.35 mL/min [12]. Our 0.2 mL/min flow rate fell within the UPLC flow rate range reported in the literature.

The solvent gradients applied to elute peptides were quite diverse. Gradients were wide (0–50% ACN, [28]), shallow (4–24% ACN, [18]), or intermediary (4–32% ACN, [7]; 3–30% ACN, [30]; 10–45% ACN, [12]). The 6–36% ACN we selected was comparable to those reported.

Reported LC total run times ranged from 30 min [7] to 100 min [18], with various intermediate durations (40 min, [12]; 60 min, [30]; 72 min, [28]). Our selected 43 min run duration fell within this range.

### 2.5. Validating the Shotgun Proteomics Method

Based on test results, the procedure we chose for our high-throughput study employed 20 mg flour, Gnd-HCl buffer, trypsin/Lys-C digestion, and LC method 6 with a bioZen column. To validate this method, we applied it to grain samples of 96 wheat lines to confirm its robustness. For normalisation purposes, a quality control (QC) sample was created by mixing all flour samples for all 96 wheat lines together and an internal standard (IS) was spiked into the tryptic digests. The blank (mobile phase A), IS, and QC sample were injected every 24 samples during the LC sequence run. The normalisation steps of the LC–MS quantitative data considered first the flour weight, the IS content, and then the LC injection order of the QC replicates.

**Figure 6:**
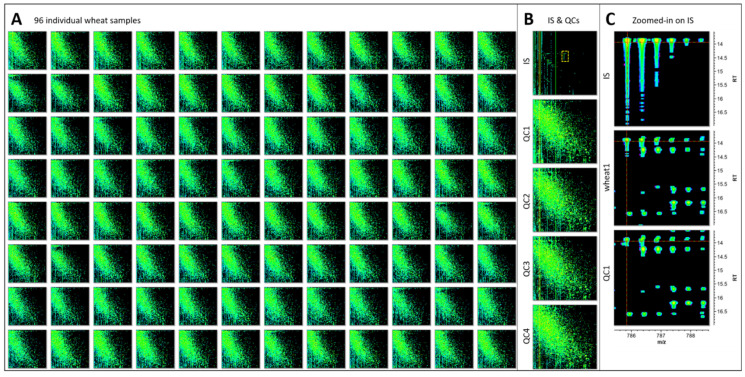
LC–MS maps for method validation. An amount of 20 mg (±0.2 mg) from 96 wheat cultivars was weighed, extracted using 0.5 mL Gnd-HCl buffer, and 10 µL extract aliquots were digested using trypsin/Lys-C. QCs and IS are described in the Materials and Methods. LC–MS data were acquired using LC method 6. (**A**) LC–MS maps of 96 individual wheat tryptic digests; (**B**) LC–MS maps of internal standard (IS) glu[1]-fibrinopeptide B and quality control samples (QCs), boxed section is where IS resolves and is zoomed-in in panel C; (**C**) zoomed-in section of LC–MS maps at 14–17 min and 785–789 *m*/*z* of the whole IS cluster on its own, in a wheat sample and in the QC sample; crossed dotted red lines pinpoint the 1st isotopic LC–MS peak of IS.

The reproducibility of the method is confirmed by the similarity of the LC–MS profiles obtained for all 96 samples (Figure 6A) and QC replicates (Figure 6B). The cluster of isotopic peaks for the IS on its own or within the QC of a wheat sample is displayed in Figure 6C. 

**Figure 7:**
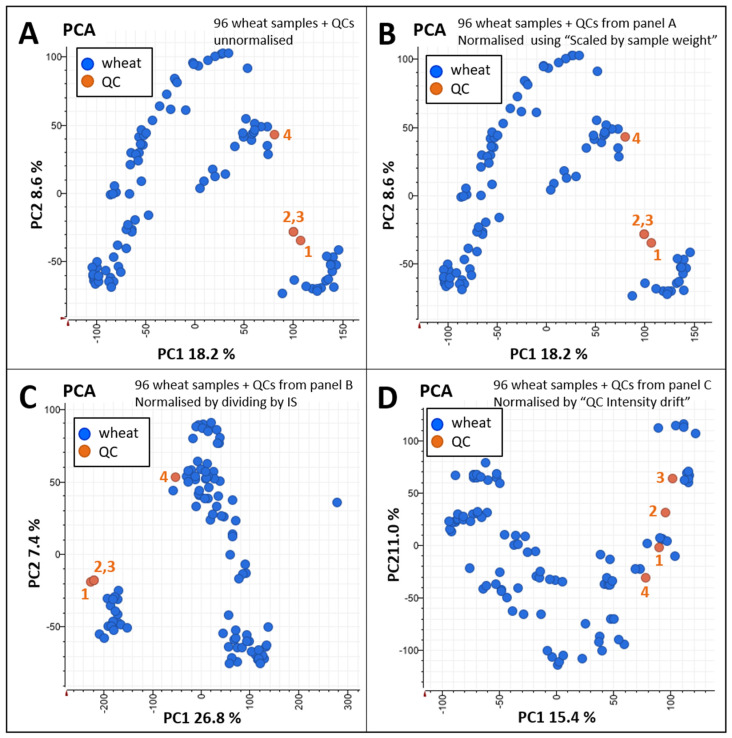
Principal component analysis (PCA) for method validation. Twenty milligrams (±0.2 mg) from 96 wheat cultivars was weighed, extracted using 0.5 mL Gnd-HCl buffer, and 10 µL extract aliquots were digested using trypsin/Lys-C. QCs and IS are described in the Materials and Methods. LC–MS data were acquired using LC method 6. (**A**) PC1 vs. PC2 plot based on unnormalised LC–MS quantitative data of the 96 wheat and QCs samples; (**B**) PC1 vs. PC2 plot based on LC–MS quantitative data from panel A normalised using the sample weights; (**C**) PC1 vs. PC2 plot based on LC–MS quantitative data from panel B normalised using the IS cluster; (**D**) PC1 vs. PC2 plot based on LC–MS quantitative data from panel C normalised using the injection order of the QCs (indicated with the orange numbers) and the ‘intensity drift’ algorithm of Genedata Analyst.

A PCA was performed to monitor the effects of the successive normalisation steps (Figure 7). 

Figure 7A presents the unnormalised quantitative data. Wheat samples distributed all over PC1 vs. PC2 across two main areas, whereas QCs were confined to a smaller region. Only three QCs were visible as two of them completely overlapped. Normalising against flour weights had no impact (Figure 7B). This proved that weighing flour with 1% accuracy successfully eliminated such technical variation. This normalisation step could potentially be omitted during our future large-scale study. Normalising against IS abundance had a noticeable effect by creating tighter groups, and thus improving data reproducibility (Figure 7C). The ultimate normalisation step based on the LC injection order using QCs had the greatest impact on the data by scattering the points more broadly across the PCA plot and reorganising QCs along PC2 (Figure 7D). There were no longer two discrete groups of wheat samples, suggesting that the normalisation successfully minimised uncontrollable technical variation.

Shotgun bottom-up proteomics involves a series of processing steps that encompass various factors which if not controlled could add significant technical variability to the quantification results to the detriment of biological variability [48]. Reducing technical variability is essential for the accurate study design and estimating statistical power. High reproducibility of a dataset is achieved with the aid of robust standard operating procedures, accurate sample weighing and volume pipetting, regular instrument maintenance, LC autosamplers, frequent mass calibration, technical replicates, ISs, and QCs. In gel-free and label-free proteomics, the intensities of the same peptides can be integrated by measuring the area or volume under LC–MS peaks, which are linearly proportional to the concentration of the peptides [49]. These quantities can then be compared across different biological states by analysis of several samples as part of a carefully designed experiment that minimises technical variation. In complex mixture analysis, because not all peptides are selected for MS fragmentation in every sample, it is critical to find and quantify the peptide in different samples, even if it has only been sequenced once [50]. This is computationally achieved by sophisticated software, such as Genedata Expressionist, which enable background noise reduction, realignment of peptides based on RT and *m*/*z,* and quantitation of extracted ion chromatograms across multiple LC–MS runs [51]. 

Normalisation methods are required to maintain data quality and allow for meaningful quantitative comparisons across multiple samples. Sample amounts are commonly used for normalisation purpose in metabolomics [52]. It is not as common in proteomics as the protein concentrations of biological matrices can be assayed. Yet such assays are costly and time consuming and as such do not fit well in large-scale high-throughput proteomics experiments that rely on cheap and rapid screening methods. QCs have been employed to normalise LC–MS data and minimise intra- and inter-sample batch differences [53] by notably correcting RT and *m*/*z* shifts [54]. IS have been used to ensure that small quantitative differences between different biological states are not missed [55]. In this study, we successfully resorted to all these normalisation strategies to correct unwanted biases.

### 2.6. Data Mining of Protein Identification

Table 1 summarises the number of LC–MS peaks, clusters, and their characteristics, along with the number of identities. 

The 60,473 LC–MS peaks detected were grouped into 20,254 clusters, comprising 2 to 11 peaks, bearing 2 to 10 positive charges, spanning a mass from 589 to 8990 Da, and displaying a dynamic range of 6 magnitude orders (intensity from 9 to 137,721). Of all the LC–MS clusters analysed, 13,165 (65%) led to an AA sequence matching to 12,404 (94%) unique peptides, which belonged to 8738 unique UniProt accessions. The majority of these accessions (5652; 65%) were uncharacterised proteins. Known protein accessions were redundant and corresponded to 1390 unique descriptions. The full list of quantified clusters and identified peptides is available in Supplementary Table S2. 

A linear model was performed to find the cultivar-responsive proteins; *p* values are listed in Supplementary Table S3. The most significant protein is an RNA-binding protein, (RRM domain-containing protein, *p* value = 1.0 × 10^−35^), followed by an uncharacterised protein (accession A0A3B6KK32, *p* value = 2.4 × 10^−34^). Other significant known proteins include alpha-amylase inhibitors, dehydrin, storage proteins (low molecular weight glutenins, avenin, globulin, gliadins), and numerous enzymes (e.g., glyceraldehyde-3-phosphate dehydrogenase, fructose-bisphosphate aldolase, peroxidase, sucrose synthase). Lakhneko and colleagues compared two Ukrainian modern wheat cultivars with a landrace genotype and found that several gliadins and glutenins were differentially expressed, as well as nongluten proteins such as trypsin/alpha-amylase inhibitor CMX2 and globulin-3A [16]. DiFrancesco and colleagues compared three Italian wheat genotypes and observed their proteomes to be very similar [18].

The UniProt Knowledgebase (UniProtKB) is the central repository for proteins, with accurate, consistent, and rich annotations pertaining to protein name or description, biological function, AA sequence, taxonomic data, and citation information [56]. Furthermore, it offers many useful tools together with links to most relevant protein databases. This is highly advantageous not only for protein identification but also for data mining purposes; this is why we used UniProt wheat accessions to build our FASTA file. 

Bread wheat is not a model species and the sequencing of its gigantic genome was an immense undertaking just recently completed [2,3]. As such, not many of the numerous online freewares available to the proteomics community can be applied to wheat datasets. The following part presents a few tools we found suitable to mine our wheat proteome. This necessitated converting the 8738 UniProt accessions into the prerequisite identifiers needed for KEGG, various gene ontology (GO) tools, and Pathway Tools via the online BreadwheatCyc interface. These identifiers can be found in Supplementary Table S2. 

The Kyoto Encyclopedia of Genes and Genomes hosts a suite of databases and associated software for understanding high-level functions and utilities of the biological system (cell to ecosystem) from molecular-level information, especially large-scale datasets generated by genome sequencing and other omics technologies [57]. KEGG has been leading the way in mapping biochemical pathways for many years [58]. The 3115 KEGG Orthology (KO) identifiers mapped onto 381 pathways, including 340 (11%) KOs from metabolic pathways. Other flagged pathways listed 188 KOs participating to the biosynthesis of secondary metabolites, 43 KOs belonging to the carbon metabolism, 37 KOs involved in AA metabolism, 42 KOs acting in the biosynthesis of cofactors, 11 KOs from the fatty acid metabolism, and, interestingly, 66 KOs linked to microbial metabolism (Supplementary Figure S2). 

As expected, many enzymes involved in starch and sucrose metabolism were identified in wheat flour, such as alpha- and beta-amylases (Supplementary Figure S3A), along with numerous storage proteins, among which are many gliadins and glutenins (Supplementary Table S2). 

The prominence of storage proteins was confirmed by the UniProtKB Retrieve/ID mapping tool which fetches the numerous annotations linked to UniProt accessions, including GO terms. Out of the 5483 molecular function (MF) GO terms found in our 8738 UniProt accessions, 225 were assigned to the nutrient reservoir activity (Supplementary Figure S4).

AgriGO v2.0 is a web-based tool and database for gene ontology analyses that specifically focuses on agricultural species [59]. It conveniently hosts user-friendly data mining tools, including the singular enrichment analysis (SEA). SEA highlighted that the nutrient reservoir and glycogen (starch) synthase activities were among the MF GO terms enriched in our data, as illustrated by the hieratical diagrams in Supplementary Figure S5A. 

This was supported by the highly significant cellular component (CC) GO term, amyloplast, as well as the enriched biological process (BP) starch-related processes (Supplementary Figure S5). Other over-represented MF categories pertained to peptidase regulator and inhibitor activities, along with alpha-amylase inhibitor activity. 

Surprisingly, KEGG mapped many of our identities to microbial metabolisms, including plant–pathogen interaction pathway (Supplementary Figure S3B). This was confirmed by the AgriGO enrichment analysis which flagged many BPs associated with response to biotic stimulus and more precisely, a defence response to fungus (Supplementary Figure S5). Indeed, several chitinases and chitin-binding type-1 domain-containing protein were identified in this work (Supplementary Table S2).

REVIGO is another user-friendly online tool that reduces and visualises gene ontologies [60]. Using a list of GO terms and their occurrences, REVIGO generates scatterplots, interactive graphs, tree maps for each of the broader class (MF, CC, BP), as well as tag clouds. Mining the 3000 unique GO terms found in our study with REVIGO produced 1467 BPs, 468 CCs, and 1043 MFs. The defence response was also featured as the most prominent BP class, followed by polysaccharide catalytic process, and translation (Supplementary Figure S6).

As an alternative to KEGG system, Pathway tools [61] offers an excellent global overview of the pathways featuring the proteins identified in this work. A drawback is that only Traes accessions could be mapped and not UniProt ones. Using BlastGUI software [62] to blast the 8738 (95%) UniProtKB accessions, we retrieved 8288 Traes identifiers, of which 1949 (24%) could be mapped in *T. aestivum* Pathway tools cellular overview (Supplementary Figure S7A). 

As previously noted, the secondary metabolite, carbohydrate, nucleoside, and nucleotide, as well as fatty acid and lipid metabolisms, were well represented. Furthermore, this type of visualisation allowed us to quickly observe that wheat flour contained many enzymes involved in hormone biosynthesis, including brassinosteroid, gibberellin, jasmonic acid, auxin, cytokinin, abscisic acid, and strigol (Supplementary Figure S7B). Hormone metabolism did not stand out when the other data visualisation tools were used (UniProt Retrieve/ID mapping, KEGG, GO hieratical diagram, and REVIGO). 

The suite of online tools used in this study helped us mine the data more in depth and efficiently highlights pathways of significance in our study, such as carbohydrates, as expected, but more surprisingly the biotic response and hormonal metabolism. Some of the tools described here have been used in other wheat proteomics studies such as KEGG [33] and GO classifications [7,18,23,63].

## 3. Materials and Methods

The experimental design is illustrated in Figure 1. Steps involving technical optimisation were weighing, protein extraction, protein digestion, and UPLC separation. 

### 3.1. Materials

#### 3.1.1. Wheat Cultivation and Sampling

A total of six wheat cultivars and four replicates were used for the optimisation tests. LRBP Flanker, LRBP Mustang, LRBP Impala, and Suntop are hard-grain, bread-quality cultivars, while QAL2000 and Sunsoft98 are soft wheat varieties used for cookies and cakes. All were sourced from wheat trials grown in 2019. 

For the validation studies, 96 globally diverse cultivars were randomly selected from a reference library grown at Horsham Victoria.

*T. aestivum* cultivars are listed in Supplementary Table S1.

#### 3.1.2. Wheat Grain Processing

For each cultivar, grains were packaged into a small plastic zip bag with a QR code label. The label was scanned into a spreadsheet to keep track of the samples. The content of the bag was transferred into a 50 mL grinding jar with two 8 mm and two 3 mm metal grinding balls. 

The jars were placed in an automated tissue homogeniser and cell lyser (Geno/Grinder^®^ 2010, SPEX SamplePrep, Metuchen, NJ, USA); 36 jars were processed simultaneously. The grains were pulverised twice for 2 min at 1500 rpm with a 15 s break in between to avoid overheating. Using a curved metal spatula, the flour was transferred from the jar into a 2 mL microtube labelled with the QR code. The empty dirty jars were reused by first soaking them into 0.5% Decon 90 detergent overnight, rinsing them, and finalising their thorough cleaning in a dishwasher fed by RO water. 

A wheat quality control (QC) sample was prepared by sampling 50 mg (±0.05 mg) from each of the 96 flour samples and mixing them all thoroughly. The microtubes were stored at −80 °C until protein extraction.

### 3.2. Methods

#### 3.2.1. Flour Weighing, Protein Extraction, and Protein Assay

Flour was weighed using a metal microspatula and a precision balance (Entris, Sartorius, Goettingen, Germany). The spatula was ethanol-wiped between samples. Three weights were tested: 10, 20, and 30 mg (±1%) using four technical replicates. The amount of flour required was transferred into a 1.5 mL microtube labelled with the corresponding QR code for protein extraction.

Two extraction buffers were tested on three wheat cultivars using four technical replicates: Gnd-HCl buffer (6 M Guanidine hydrochloride, 0.1 M Bis-Tris, 10 mM DTT, 5.37 mM sodium citrate tribasic dihydrate) and a urea buffer (6 M Urea, 0.1 M Bis-Tris, 10 mM DTT, 5.37 mM sodium citrate tribasic dihydrate). A 0.5 mL volume of extraction buffer (either Gnd-HCl or urea) was added to the 10, 20, and 30 mg flour. The flour was dissolved using a MS 1.5 sonicator probe (Ultrasonic Homogeniser SONOPULS mini 20, Bandelin, Berlin, Germany) for 30 s with 90% amplitude. Only one tube could be probe-sonicated at a time. The probe was cleaned in between samples by probe-sonicating in milliQ water for 5 s with 90% amplitude and wiping it with a fibre-free wipe. The tubes were briefly vortexed and incubated for 60 min in a thermoblock (Digital Dry Bath/Block Heater, Thermo Scientific, Scoresby, VIC, Australia) either at 60 °C (for Gnd-HCl samples) or 35 °C (for urea samples). 

The tubes were left to cool to room temperature for 5 min and 10 µL of 1 M iodoacetamide was added to each tube. The tubes were thoroughly mixed for 30 s using a vortex mixer (MTV1 Multi Tube Vortex Mixer, Ratek, Boronia, VIC, Australia) at high speed and left to incubate at room temperature in the dark for 30 min.

The tubes were then centrifuged using a benchtop centrifuge (5415D Digital Microfuge, Eppendorf, Macquarie Park, NSW, Australia) at 13,000 rpm for 15 min at room temperature. The supernatant was transferred into a fresh 1.5 mL microtube labelled with the QR code and stored at −80 °C until protein digestion.

The protein content was measured using a BCA protein assay (Pierce, ThermoFisher Scientific, Scoresby, VIC, Australia) and BSA as a standard as per the manufacturer’s instructions.

#### 3.2.2. Protein Digestion, Digest SPE Clean-Up, and Peptide Reconstitution

Three sets of orthogonal proteases were tested in parallel: Glu-C (V1651, Promega, Alexandria, NSW, Australia), chymotrypsin (V1062, Promega, Alexandria, NSW, Australia), and a trypsin/Lys-C mix (V5078, Promega, Alexandria, NSW, Australia). Each set of frozen lyophilized proteases was resuspended using 50 mM ammonium bicarbonate immediately prior to use.

Four technical replicates were used. An extract volume corresponding to 100 µg of proteins was used for the digestions and diluted 6 times with 50 mM ammonium bicarbonate to drop the molarity of the chaotrope reagents to 1 M. One microgram of enzyme (either Glu-C, chymotrypsin, or trypsin/Lys-C) was added to the protein aliquot to reach a ratio of 1:100 protease:protein. Tubes were left to incubate overnight (18 h) at room temperature for chymotrypsin, and in an oven (oven APS 60 L, ThermoFisher Scientific, Scoresby, VIC, Australia) at 37 °C for Glu-C and trypsin/Lys-C. The digestion reaction was stopped by adding 10% formic acid (FA) to a final concentration of 1%. The internal standard (IS, [Glu^1^]-fibrinopeptide B human, F3261, Sigma, Port Melbourne, VIC, Australia) was added at a final concentration of 1 µg. 

Protein digests were cleaned using 96-wells SPE plates (Strata C18-E 100 mg P/N 8E-S001-EGB, Phenomenex, Lane Cove, NSW, Australia) and a plate manifold (96-Well Plate Manifold, Universal, with Vacuum Gauge, Phenomenex, Lane Cove, NSW, Australia) fitted to a vacuum tap.

Each well of the plates was primed first with 1 mL 80% acetonitrile (ACN/) 0.1% FA/H_2_O, then with 1 mL 0.1% FA/H_2_O prior to being loaded with the digests. Digests were desalted with 1 mL 0.1% FA/H_2_O and eluted with 250 µL 80% ACN/0.1% FA/H_2_O into a fresh collection plate (350 µL Strata 96-well collection plate, Phenomenex, Lane Cove, NSW, Australia). The collection plates were sealed with a silicone lid and were stored at −80 °C until evaporation.

Collection plates were placed into a vacuum centrifuge (SPD-2010 SpeedVac, ThermoFisher Scientific, Scoresby, VIC, Australia) without heat overnight until complete evaporation of the eluates. Peptide digests were reconstituted by adding 70 µL of 0.1% FA/H_2_O to each well. The digests were dissolved by shaking the plates for 50 min at medium speed using a vortex mixer (MTV1 Multi Tube Vortex Mixer, Ratek, Boronia, VIC, Australia) at room temperature. The collection plates were sealed with a silicone lid and were stored at −80 °C until further use.

The collections plates were briefly spun for 30 s in a plate centrifuge with a swing-out rotor (3–16 L tabletop centrifuge, Sigma, Port Melbourne, VIC, Australia) at minimum speed (50 rpm) to pool the entire reconstituted sample at the bottom of the wells.

#### 3.2.3. LC–MS and LC–MS/MS

##### LC Separation Columns

We tested two RP–LC columns in this study: an Aeris column (Aeris 1.7 um Peptide XB-C18, 100 Å, LC column 150 mm × 2.1 mm, Phenomenex, Lane Cove, NSW, Australia) and a bioZen column (bioZen 1.7 um Peptide XB-C18, 100 Å, LC column 150 mm × 2.1 mm, Phenomenex, Lane Cove, NSW, Australia). 

Both columns were designed for peptide separation and contained core-shell silica. The Aeris series are well-established columns that we have used successfully for many years [37,39,41,42,64].

The bioZen series was released a few years ago and differed from the Aeris columns in their titanium hardware with minimum priming. We also have used them successfully on a recent project on cannabis [38,40,42,43]. 

We never before formally compared these two columns; however, we did optimise chromatograms in previous works and found that elevated oven temperature (60 °C and above) improved peptide peak separation [39].

##### LC Methods

Several chromatographic methods were tested with the ultimate objective to compromise between speed of analysis and LC–MS peak resolution. The UHPLC system used was a Vanquish Flex Binary UHPLC System (Vanquish UHPLC+ focused, ThermoFisher Scientific, Scoresby, VIC, Australia).

Mobile phase A was 0.1% FA/H_2_O and mobile phase B was 0.1% FA/ACN. The needle was solution was 80% isopropanol/H_2_O and the rear seal wash solution was 10% isopropanol/H_2_O. The needle wash solution was 10% isopropanol/H_2_O. The needle was washed after each injection. Blanks were injected from a 10 mL vial containing 0.1% FA/H_2_O. 

LC method 1: 0.1 mL/min flow rate, 60 min LC run duration, 3% B for 6 min, 3–40% B gradient for 33 min, 40–90% B gradient for 1 min, 90% B for 15 min, drop down to 3% B in 30 sec, 3% B for 4.5 min. 

LC method 2: 0.1 mL/min flow rate, 45 min LC run duration, 3% B for 6 min, 3–11% B gradient for 1 min, 11–40% B gradient for 31 min, 40–90% B gradient for 1 min, 90% B for 1 min, drop down to 3% B in 30 sec, 3% B for 4.5 min.

LC method 3: 0.2 mL/min flow rate, 45 min LC run duration, 3% B for 6 min, 3–15% B gradient for 1 min, 15–40% B gradient for 31 min, 40–90% B gradient for 1 min, 90% B for 1 min, drop down to 3% B in 30 sec, 3% B for 4.5 min.

LC method 4: 0.2 mL/min flow rate, 45 min LC run duration, 3% B for 2.5 min, 3–40% B gradient for 35.5 min, 40–90% B gradient for 1 min, 90% B for 1 min, drop down to 3% B in 30 sec, 3% B for 4.5 min.

LC method 5: 0.2 mL/min flow rate, 38 min LC run duration, 6% B for 2.5 min, 6–36% B gradient for 30.5 min, increased up to 98% B gradient for 0.1 min, 98% B for 1 min, drop down to 3% B in 0.1 min, 6% B for 3 min.

LC method 6: 0.2 mL/min flow rate, 38 min LC run duration, 6% B for 2.5 min, 6–36% B gradient for 30.5 min, increased up to 98% B gradient for 0.1 min, 98% B for 5 min, drop down to 3% B in 0.1 min, 6% B for 5 min. No online desalting step was applied in this method.

##### ESI–MS

The UHPLC was online with an Orbitrap Velos hybrid ion trap–Orbitrap mass spectrometer (ThermoFisher Scientific, Scoresby, VIC, Australia) fitted with a heated electrospray ionisation (HESI) source. The instrument was mass calibrated weekly.

HESI parameters were: needle at 3.9 kV, 100 µA, sheath gas flow 20, auxiliary gas flow 7, sweep gas flow 2, source heated to 200 °C, capillary heated to 275 °C, and S-Lens RF level 55%. 

When online desalting was applied (LC methods 1–5), for the first 6 or 2.5 min of the LC run, the UHPLC flow was sent to waste using a divert valve, then switched to source for the remainder of the run and finally reverted back to waste for the last minute of the run during the LC equilibration phase. Spectra were acquired using the full MS scan mode of the Fourier transform (FT) orbitrap mass analyser in positive ion mode at a resolution of 15,000 along a 300–2000 *m*/*z* mass window in profile mode with 3 microscans. For improved quantitation, these parameters minimised the duty cycle and therefore maximised the number of data points collected across LC–MS peaks [65,66].

##### ESI–MS/MS

Tandem mass spectrometry was applied to all the samples that underwent protein extraction and digestion optimisation and LC method 1 (60 min LC run).

The HESI and full scan MS parameters were as described above. 

Using the Nth order double play method, MS/MS spectra were acquired in data-dependent mode. Singly charged peptides were ignored. In the linear ion trap, the 10 most abundant peaks with charge state >2 and a minimum signal threshold of 3000 were fragmented using collision-induced dissociation (CID) with a normalised collision energy of 35%, 0.25 activation Q, and activation time of 10 ms. The precursor isolation width was 2 *m*/*z*. Dynamic exclusion was activated, and peptides selected for fragmentation more than once within 30 s were excluded from selection for 180 s.

##### LC–MS Validation Run

The chosen extraction, digestion, and LC–MS methods were validated on 96 randomised wheat samples listed in Supplementary Table S1. 

Blank, IS, and QC samples were injected at the start of the sequence run and every 24 samples.

#### 3.2.4. Data Processing, Database Search, and Statistical Analyses

##### Data File Processing

BPCs in Figure 5 were created using Xcalibur Qual Browser software (ThermoFisher Scientific, Scoresby, VIC, Australia). 

The LC–MS and LC–MS/MS data files were processed in the Refiner MS module of Genedata Expressionist^®^ 13.0 (Genedata AG, Basel, Switzerland) as described in [40,42,43]. The visualisation 2-D mapping tool of Refiner was used to produce the LC–MS maps throughout this article. LC–MS peaks belonging to the same isotopic profile are grouped into clusters whose integrated volumes are exported for statistical analyses.

##### Protein Identification

Database searching of the LC–MS/MS.RAW files was performed in Proteome Discoverer (PD, ThermoFisher Scientific, Scoresby, VIC, Australia) 1.4. All 142,969 *T. aestivum* protein sequences publicly available on 26 February 2020 from UniProtKB (https://www.uniprot.org/uniprot/?query=triticum%20aestivum&fil=organism%3A%22Triticum+aestivum+%28Wheat%29+%5B4565%5D%22&sort=score) were downloaded as a FASTA file (accessed on 26 February 2020). The FASTA protein sequences were imported and indexed in PD 1.4 and Mascot. 

Both SEQUEST and Mascot algorithms were used to search the indexed FASTA file. The database searching parameters specified trypsin/Lys-C, chymotrypsin, or Glu-C as the digestion enzyme and allowed maximum number of missed cleavages (12 for SEQUEST and 9 for Mascot). The benefit of allowing for a high number of missed cleavages has been extensively discussed in Vincent et al. 2019 [40]. The precursor mass tolerance was set at 10 ppm and fragment mass tolerance set at 0.8 Da. The peptide absolute Xcorr threshold was set at 0.4 and protein relevance threshold was set at 1.5. Carbamidomethylation (C) was set as a static modification. Guanidylation (K, and N-terminus) was set as a dynamic modification. The target decoy peptide-spectrum match (PSM) validator was used to estimate false discovery rates (FDR). At the peptide level, peptide confidence value set at high was used to filter the peptide identification, and the corresponding FDR on peptide level was less than 1%. 

Quantitative data and identification results are in Supplementary Table S2. LC–MS raw files are available from the MassIVE public repository (https://massive.ucsd.edu/ProteoSAFe/static/massive.jsp, MSV000088253 (accessed on 26 February 2020)).

##### Data Normalisation and Statistical Analyses

Statistical analyses were performed using the Analyst module of Genedata Expressionist^®^ 13.0 (Genedata AG, Basel, Switzerland) where columns denote plant samples and rows denote digested peptides. 

For the method validation dataset containing 96 wheat samples and 4 QCs, 3 successive normalisation steps were computed. First, the flour weights (20 ±0.2 mg) were used with the ‘Sample Weight Scaling’ algorithm; second, the IS cluster was used with the ‘Reference Row’ algorithm with the averaging method ‘Arithmetic Mean’ and the relative function ‘Divide’; finally, the QCs were used with the ‘Intensity Drift’ algorithm and the LC injection order of the samples.

PCAs were performed on rows using a covariance matrix with 50% valid values and row mean as imputation. A linear model was applied using y = a, where a is the fixed factor cultivar.

The quantitative data of the identified peptides were exported to Microsoft Excel 2016 (Office 365) spreadsheet and plotted as line charts and histograms. The Excel functions AVERAGE and STDEV were used to plot the histograms. The Excel function COUNT was used to compute the frequency of the peptides in the samples across extraction and digestion methods; the Venn diagrams were drawn in Microsoft Powerpoint 2016 (Office 365).

##### Data Mining

The Retrieve/ID mapping tool of UniProtKB (https://www.uniprot.org/uploadlists/ (accessed on 26 February 2020)) was used with the list of 8738 accessions identified in this study to obtain FASTA sequences, E.C. number, pathway, and GO terms (Supplementary Table S2). UniProt accessions were searched in the Poaceae page of AgriGO v2 (http://systemsbiology.cau.edu.cn/agriGOv2/ (accessed on 26 February 2020)) using the Singular Enrichment Analysis (SEA) online tool [59,67]. The Uniprot FASTA sequences were searched in AgBase-GOanna (https://agbase.arizona.edu/cgi-bin/tools/GOanna.cgi (accessed on 26 February 2020)) to retrieve agronomy-related GO annotations [68]. All the GO terms detected in this study and their frequencies were compiled into unique terms using Excel pivot tables. The REVIGO tool (http://revigo.irb.hr/ (accessed on 26 February 2020)) was used to summarise the 3000 unique GO terms using the Resnik normalised method and the ‘Tiny (0.4)’ list setting for maximum reduction [60].

The 8738 UniProt FASTA sequences were also used to retrieve KEGG ORTHOLOGY (KO) identifiers using the Assign KO tool (https://www.kegg.jp/kegg/mapper/assign_ko.html (accessed on 26 February 2020)) and specifying the Poaceae family. KO identifiers were then mapped using the KEGG Mapper Reconstruct tool (https://www.genome.jp/kegg/mapper/reconstruct.html (accessed on 26 February 2020)) [69].

The 8738 UniProt FASTA sequences were blasted against the 133,346 Traes accessions [2] hosted by the EnsemblPlants *T. aestivum* (http://ftp.ebi.ac.uk/ensemblgenomes/pub/release-51/plants/fasta/triticum_aestivum/pep/ (accessed on 26 February 2020)) using BlastGUI [62] and an e-value < 1 × 10^−6^. The other BlastGUI parameters were: threads = 4, outfmts = 6, other cmd = _max_target_seqs_1. The percentage of identity between UniProt and Traes sequences is indicated in Supplementary Table S2. The Pathway Tools software [61] was run online via the BreadwheatCyc database (https://pmn.plantcyc.org/organism-summary?object=BREADWHEAT (accessed on 26 February 2020)) using the Omics Dashboard (https://pmn.plantcyc.org/dashboard/dashboard-intro.shtml (accessed on 26 February 2020)) and the Cellular Overview tools (https://pmn.plantcyc.org/overviewsWeb/celOv.shtml?orgid=BREADWHEAT (accessed on 26 February 2020)) to map the Traes accessions.

## 4. Conclusions

We devised a high-throughput proteomics shotgun LC–MS method suitable for screening thousands of wheat flour samples. Our various tests indicated that a weight of 20 mg could be fully resuspended in a 0.5 mL volume of extraction solution. Urea and Gnd-HCl buffers yielded similar results, yet we recommend Gnd-HCl, which is less expensive. Using three sets of orthogonal proteases helped to explore in depth the wheat proteome, and the reader is encouraged to use them all. Yet, if time and money are constraints and only one protease is to be used, we recommend using the trypsin/Lys-C commercial mixture. The LC method we selected applied a 6–36% ACN gradient for 33 min. Essential aspects of the workflow were the inclusion of IS and QCs to ensure reproducibility and robustness of the method over time. Many data mining tools are available online; the ones we tried (KEGG, UniProtKB, AgriGO, REVIGO, and Pathway Tools) allowed for rapid and powerful exploration of the data under different angles, thus not only confirming the presence of the expected storage proteins and associated enzyme but also highlighting unsuspected results.

## Figures and Tables

**Table 1:** Summary of wheat grain proteome.

Items Quantified	Quantities
Number of LC–MS peaks	60,473
Number of LC–MS clusters	20,254
Cluster size range	2–11
Cluster charge range	2–10
Cluster *m*/*z* range	300.17–1996.52
Cluster mass range	598.34–8989.81
Base peak range	9–137,721
Number of clusters with peptide identity	13,165
Number of identified unique peptides	12,404
Number of identified accessions	8738
Number of identified annotated proteins	1390
Range of peptides/accession	1–65

## Supplementary Materials

The following are available online at https://www.mdpi.com/article/10.3390/ijms23020713/s1.
