# Supplementary material for: Mining the Wheat Grain Proteome
Source: Int J Mol Sci. 2022 Jan 10;23(2):713. doi: 10.3390/ijms23020713 (PMC8775872; doi:10.3390/ijms23020713)
Supplement: Supplementary file 1 [file ijms-23-00713-s001.zip › Vincent et al_wheat-proteomics-method_IJMS_Suppl Figures_revised.pptx]

## Slide 1
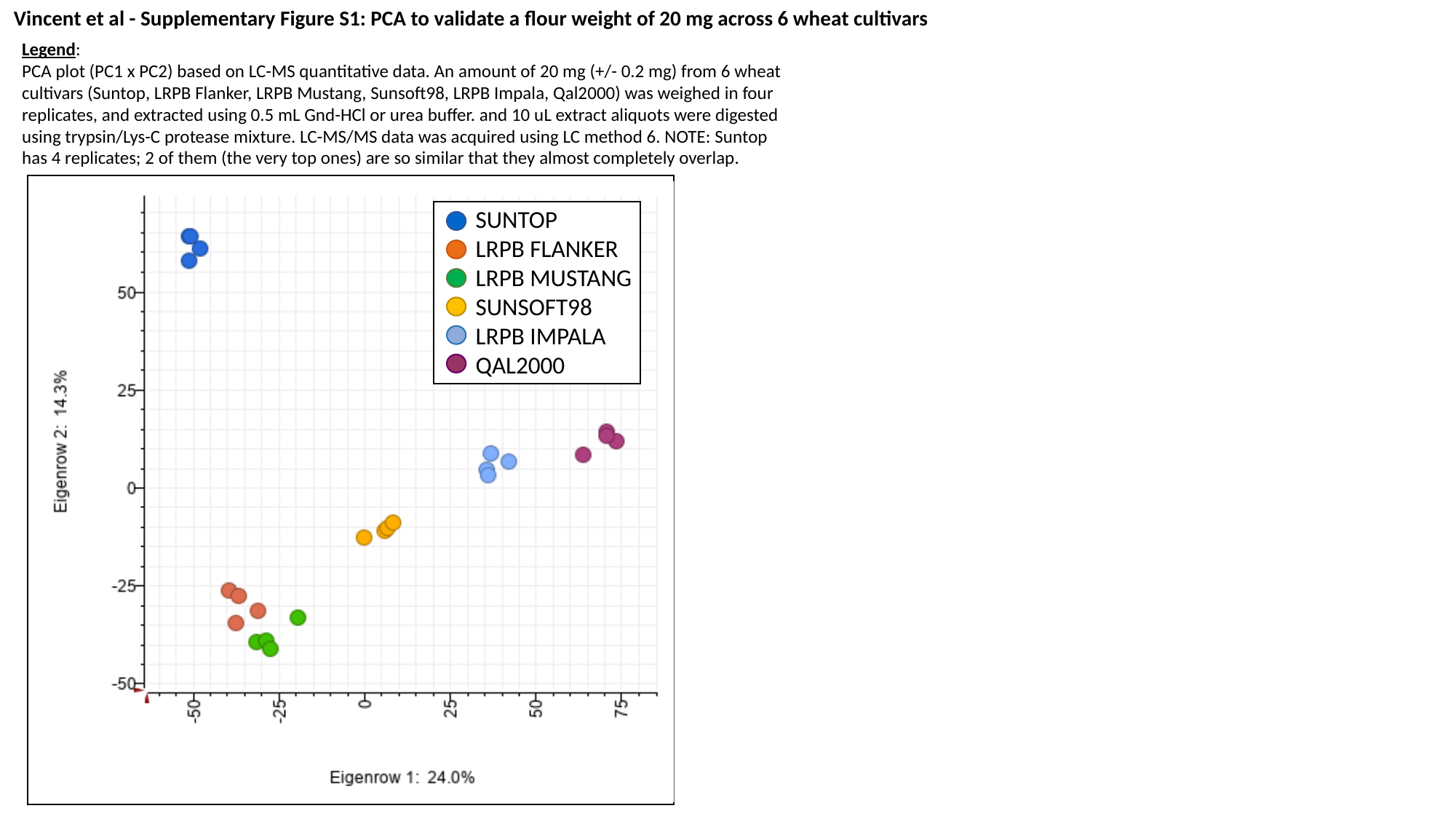

Vincent et al - Supplementary Figure S1: PCA to validate a flour weight of 20 mg across 6 wheat cultivars
Legend:
PCA plot (PC1 x PC2) based on LC-MS quantitative data. An amount of 20 mg (+/- 0.2 mg) from 6 wheat cultivars (Suntop, LRPB Flanker, LRPB Mustang, Sunsoft98, LRPB Impala, Qal2000) was weighed in four replicates, and extracted using 0.5 mL Gnd-HCl or urea buffer. and 10 uL extract aliquots were digested using trypsin/Lys-C protease mixture. LC-MS/MS data was acquired using LC method 6. NOTE: Suntop has 4 replicates; 2 of them (the very top ones) are so similar that they almost completely overlap.
SUNTOP
LRPB FLANKER
LRPB MUSTANG
SUNSOFT98
LRPB IMPALA
QAL2000

## Slide 2
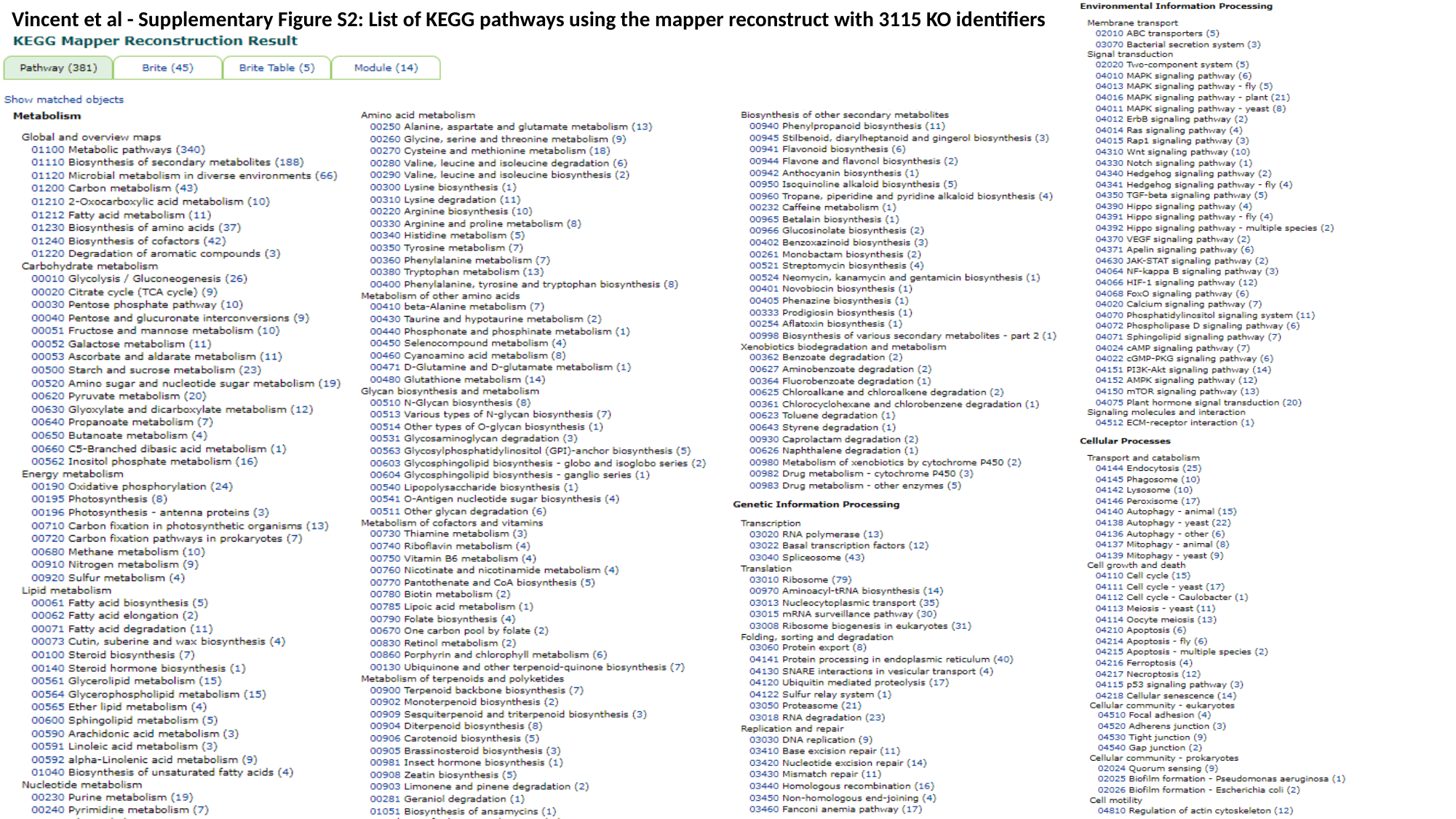

Vincent et al - Supplementary Figure S2: List of KEGG pathways using the mapper reconstruct with 3115 KO identifiers

## Slide 3
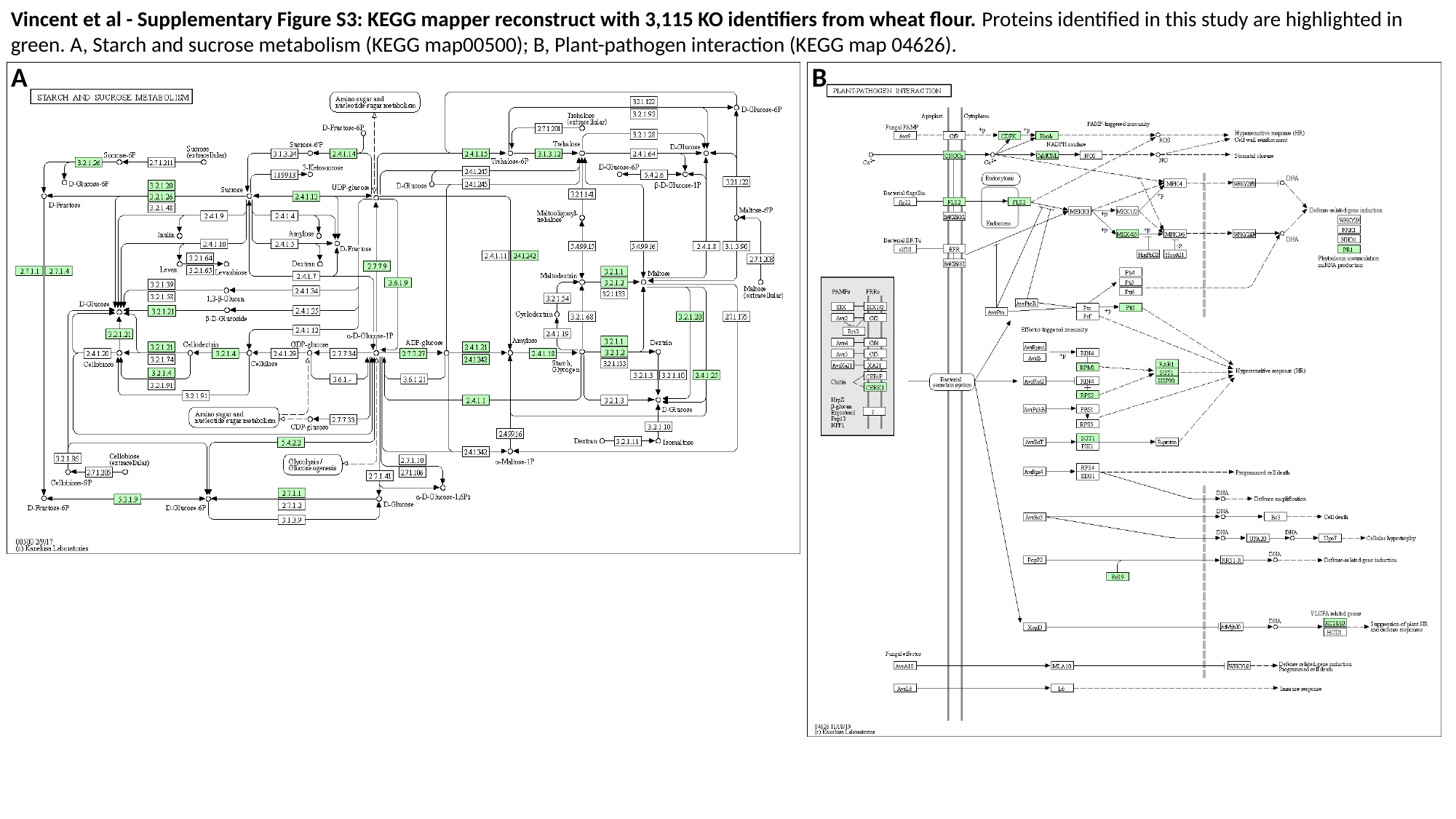

Vincent et al - Supplementary Figure S3: KEGG mapper reconstruct with 3,115 KO identifiers from wheat flour. Proteins identified in this study are highlighted in green. A, Starch and sucrose metabolism (KEGG map00500); B, Plant-pathogen interaction (KEGG map 04626).
A
B

## Slide 4
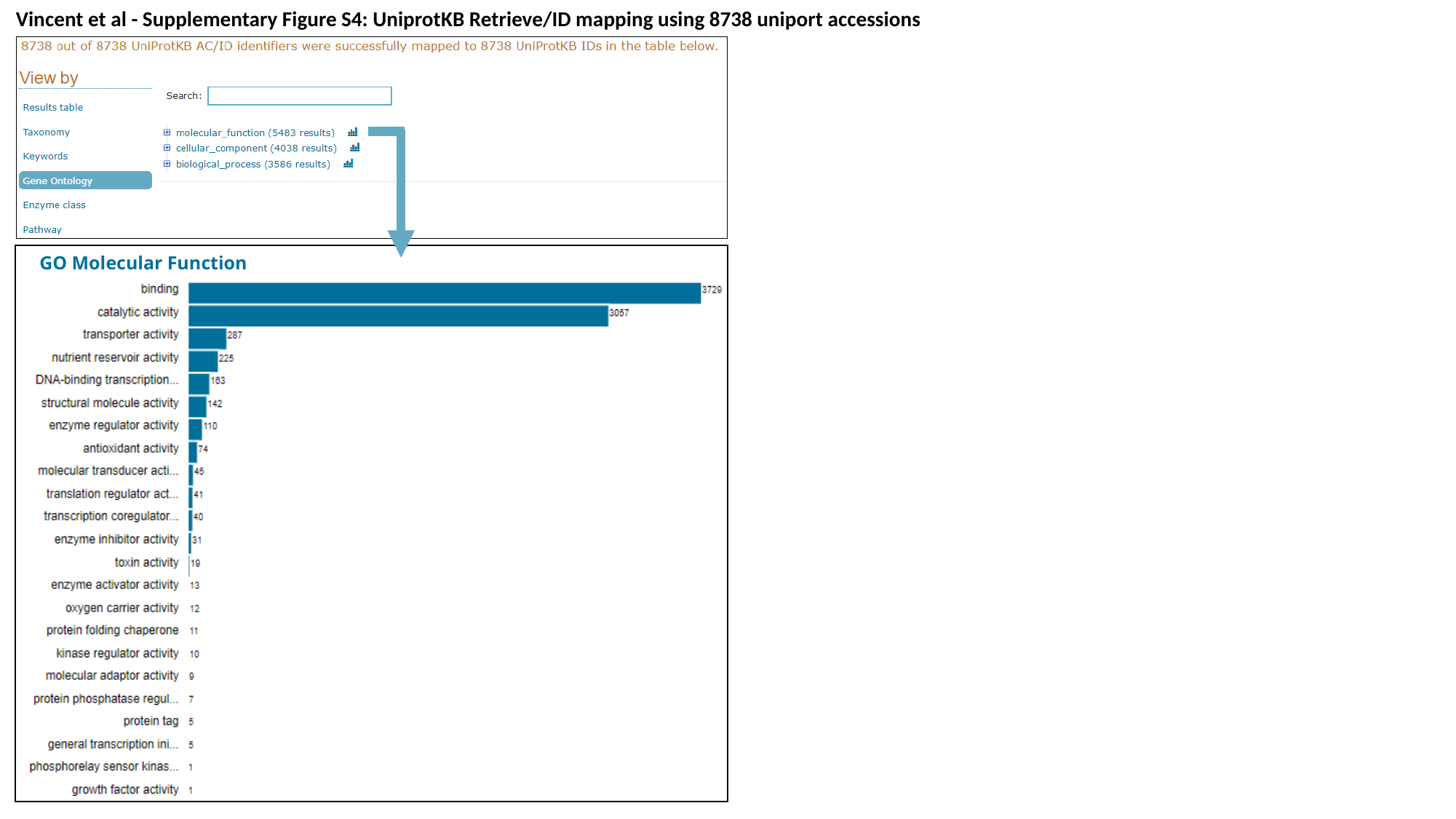

Vincent et al - Supplementary Figure S4: UniprotKB Retrieve/ID mapping using 8738 uniport accessions
GO Molecular Function

## Slide 5
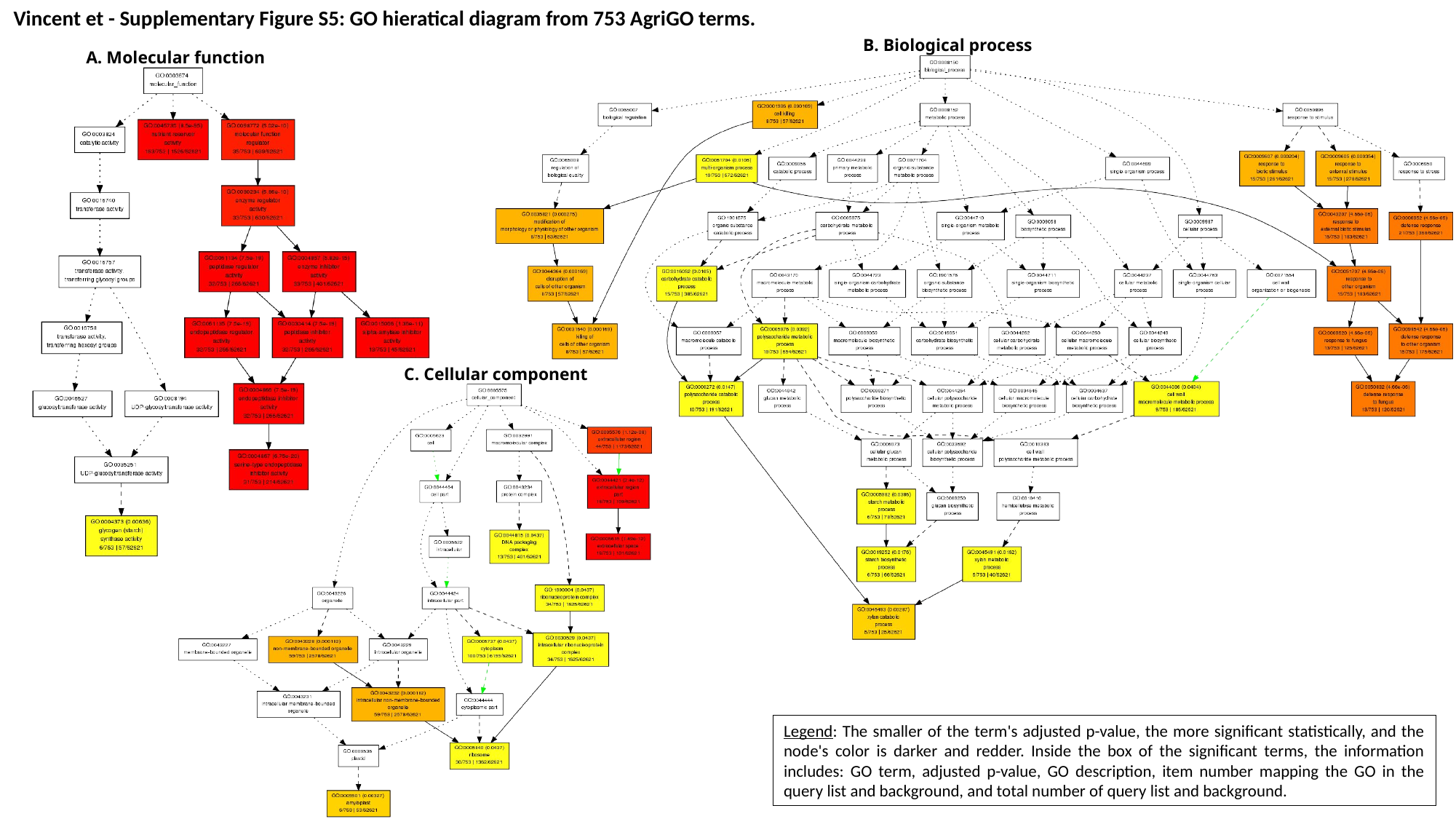

Vincent et - Supplementary Figure S5: GO hieratical diagram from 753 AgriGO terms.
B. Biological process
A. Molecular function
C. Cellular component
Legend: The smaller of the term's adjusted p-value, the more significant statistically, and the node's color is darker and redder. Inside the box of the significant terms, the information includes: GO term, adjusted p-value, GO description, item number mapping the GO in the query list and background, and total number of query list and background.

## Slide 6
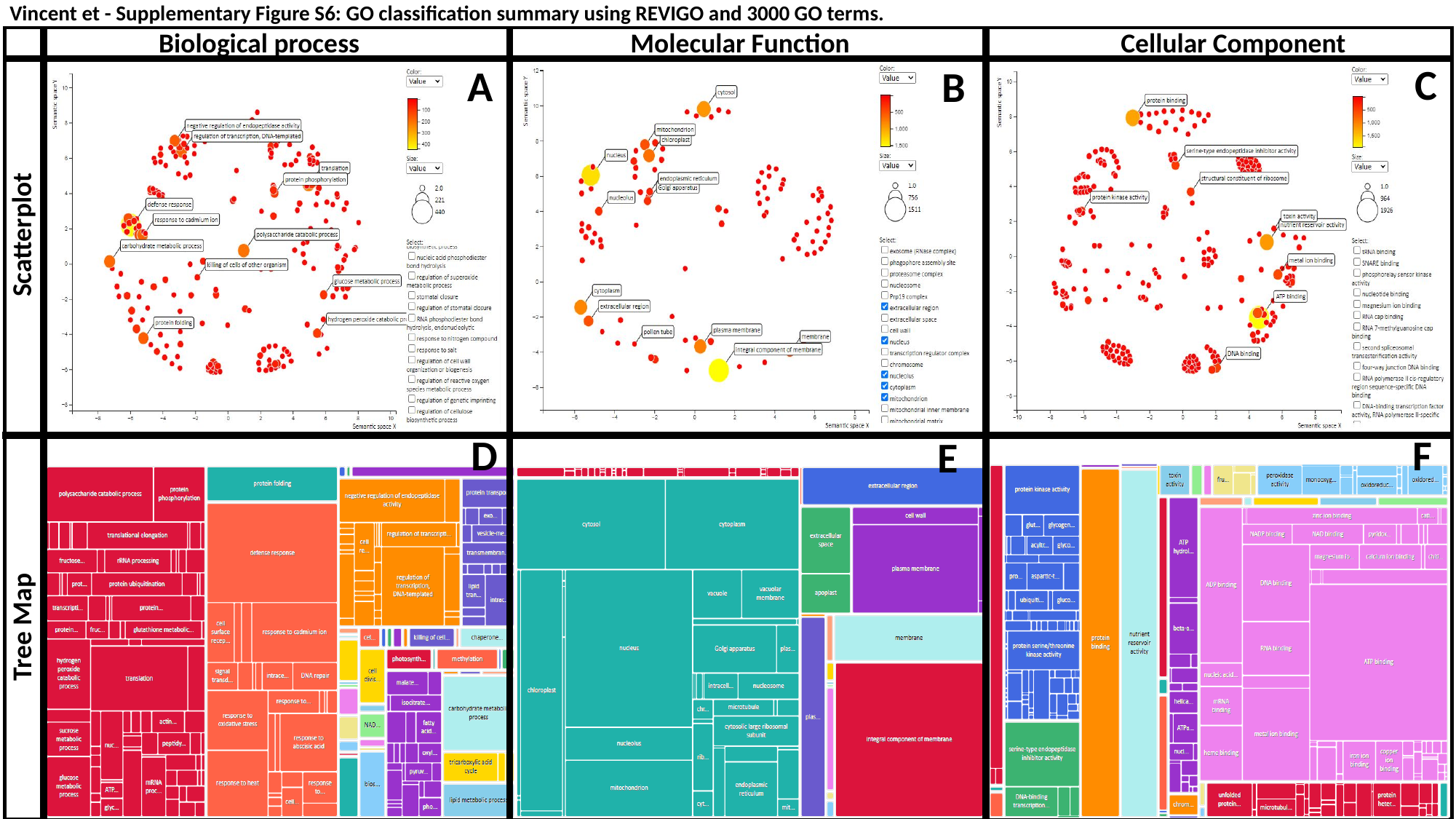

Vincent et - Supplementary Figure S6: GO classification summary using REVIGO and 3000 GO terms.
Biological process
Molecular Function
Cellular Component
C
A
B
Scatterplot
F
D
E
Tree Map

## Slide 7
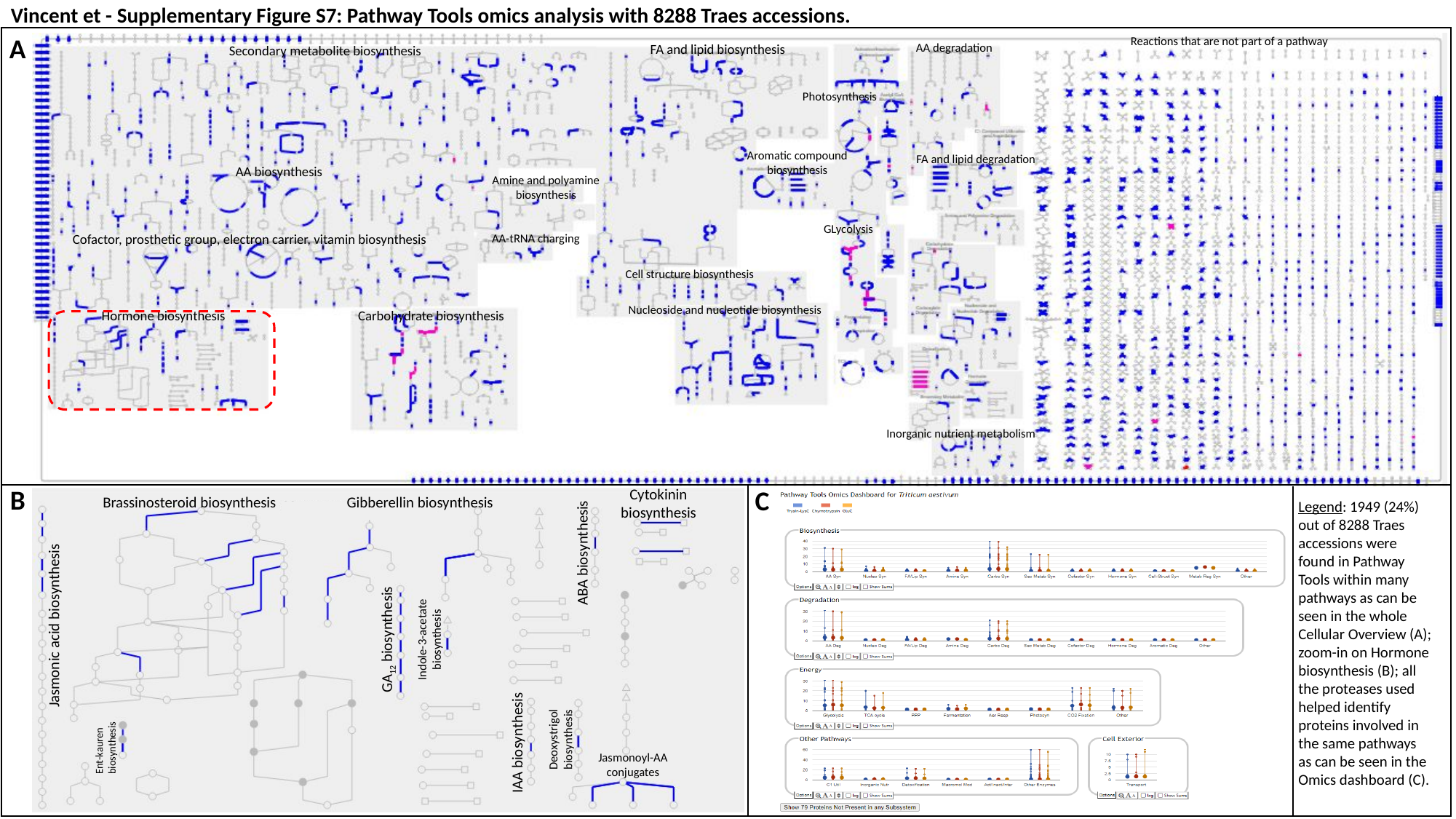

Vincent et - Supplementary Figure S7: Pathway Tools omics analysis with 8288 Traes accessions.
A
Reactions that are not part of a pathway
AA degradation
FA and lipid biosynthesis
Secondary metabolite biosynthesis
Photosynthesis
Aromatic compound biosynthesis
FA and lipid degradation
AA biosynthesis
Amine and polyamine biosynthesis
GLycolysis
Cofactor, prosthetic group, electron carrier, vitamin biosynthesis
AA-tRNA charging
Cell structure biosynthesis
Nucleoside and nucleotide biosynthesis
Hormone biosynthesis
Carbohydrate biosynthesis
Inorganic nutrient metabolism
B
C
Cytokinin biosynthesis
Brassinosteroid biosynthesis
Gibberellin biosynthesis
ABA biosynthesis
Jasmonic acid biosynthesis
Indole-3-acetate biosynthesis
GA12 biosynthesis
Deoxystrigol biosynthesis
IAA biosynthesis
Ent-kauren biosynthesis
Jasmonoyl-AA conjugates
Legend: 1949 (24%) out of 8288 Traes accessions were found in Pathway Tools within many pathways as can be seen in the whole Cellular Overview (A); zoom-in on Hormone biosynthesis (B); all the proteases used helped identify proteins involved in the same pathways as can be seen in the Omics dashboard (C).
